# Comparison of Nonexposed Endoscopic Wall-Inversion Surgery with Endoscopic-Navigated Laparoscopic Wedge Resection for Gastric Submucosal Tumours: Results of a Two-Centre Study

**DOI:** 10.1155/2019/7573031

**Published:** 2019-07-01

**Authors:** Jan Hajer, Lukáš Havlůj, Petr Kocián, Günther Klimbacher, Andreas Shamiyeh, Robert Gürlich, Adam Whitley

**Affiliations:** ^1^Second Department of Internal Medicine, Third Faculty of Medicine, Charles University and Faculty Hospital Kralovske Vinohrady, Prague, Czech Republic; ^2^Department of Surgery, Third Faculty of Medicine, Charles University and Faculty Hospital Kralovske Vinohrady, Prague, Czech Republic; ^3^Department of Surgery, Second Faculty of Medicine, Charles University and Motol University Hospital, Prague, Czech Republic; ^4^Department of Visceral Surgery, Kepler University Hospital, Linz, Austria; ^5^Department of Anatomy, Second Faculty of Medicine, Charles University, Prague, Czech Republic

## Abstract

**Introduction:**

The aim of this study was to compare the indications, operative details, and clinical outcomes of nonexposed endoscopic wall-inversion surgery with endoscopic-navigated laparoscopic wedge resection of gastric submucosal tumours.

**Methods:**

Medical records were reviewed for patients who underwent nonexposed endoscopic wall-inversion surgery (NEWS) at the Faculty Hospital Kralovske Vinohrady and endoscopic-navigated laparoscopic wedge resection (LWR) at the Kepler University Hospital. Demographic, tumour, surgical, perioperative, and follow-up data were collected and compared.

**Results:**

Eleven patients underwent NEWS and twelve patients underwent LWR. NEWS was associated with a longer operating time and more frequent suture line bleeding (3 cases in the NEWS group versus 1 case in the LWR group). Negative resection margins were achieved in all NEWS procedures and in 11 of the LWRs. The difference in size between the tumour and the resected specimen was smaller in the NEWS group. Length of hospitalisation was similar between the two groups (NEWS = 6.8 days, LWR = 6.5 days). Follow-up gastroscopies at 12 months postoperatively revealed no signs of recurrence in any of the patients.

**Conclusion:**

Nonexposed endoscopic wall-inversion surgery is a new technique for the treatment of gastric tumours. It allows for more precise resections with more frequent achievement of negative resection margins than LWRs. Additionally, it may allow for better preservation of gastric function and limit communication between the gastric lumen and peritoneal cavity. The longer operating time and more frequent complications associated with the NEWS reflects the limited experience with these new techniques.

## 1. Introduction

The aim of this study was to compare the indications, operative details and clinical outcomes of nonexposed endoscopic wall-inversion surgery (NEWS) with endoscopic-navigated laparoscopic wedge resection (LWR) for submucosal gastric tumours.

Laparoscopic wedge resection is currently the method of choice for the resection of benign and semi-malignant gastric tumours. It is superior to open resections in that it is associated with a shorter convalescence time, shorter operation length, and smaller intraoperative blood loss [1]. Additionally, there are some reports that laparoscopic resections have better survival and lower recurrence rates [2]. Laparoscopic wedge resection often requires endoscopic navigation, especially for endophytically growing tumours. In such cases, an endoscopic light can shine through the wall of the stomach and aid the surgeon in the resection.

Laparoscopic-endoscopic hybrid wedge resections differ from LWRs in that the endoscopist has a more active role. In these techniques, both the endoscopist and the laparoscopist are involved in the resection of the tumour. In this study, we used nonexposed endoscopic wall-inversion surgery, which is based on the “close first, cut later” principle that avoids creating communication between the gastric lumen and peritoneal cavity [3]. We hypothesise that this nonexposure hybrid technique is more precise, achieves negative resection margins more frequently, and better preserves the functionality of the stomach than laparoscopic wedge resection.

## 2. Methods

### 2.1. Patient Selection, Data Collection, and Settings

Medical data for all patients undergoing NEWS at the Department of Surgery of the Faculty Hospital Kralovske Vinohrady in Prague were recorded in a prospective database. This database was reviewed to find all submucosal gastric tumours between February 2016 and October 2017. Patients with endoluminally growing submucosal tumours and early gastric cancer were indicated to undergo NEWS. For the purpose of the study, to make two comparable groups, we selected only submucosal tumours; patients with early gastric cancer were excluded from the study. Medical records from the Department of Visceral Surgery of Kepler University Hospital in Linz between August 2009 and October 2017 were retrospectively reviewed to find all patients who underwent LWR of submucosal gastric tumours. Data collected from each patient consisted of demographic details (age, sex, BMI, and clinical presentation), tumour characteristics (location, size, and histology), and surgical and perioperative details (type and length of operation, complications and length of postoperative hospital stay). Gastrointestinal stromal tumours (GISTs) were classified on the basis of their mitotic index and size according to the criteria of the NIH consensus statement [4]. Patients were routinely followed up one week after discharge and underwent follow-up gastroscopy at three, six, and twelve months after discharge.

### 2.2. Nonexposed Endoscopic Wall-Inversion Surgery

**Figure 1:**
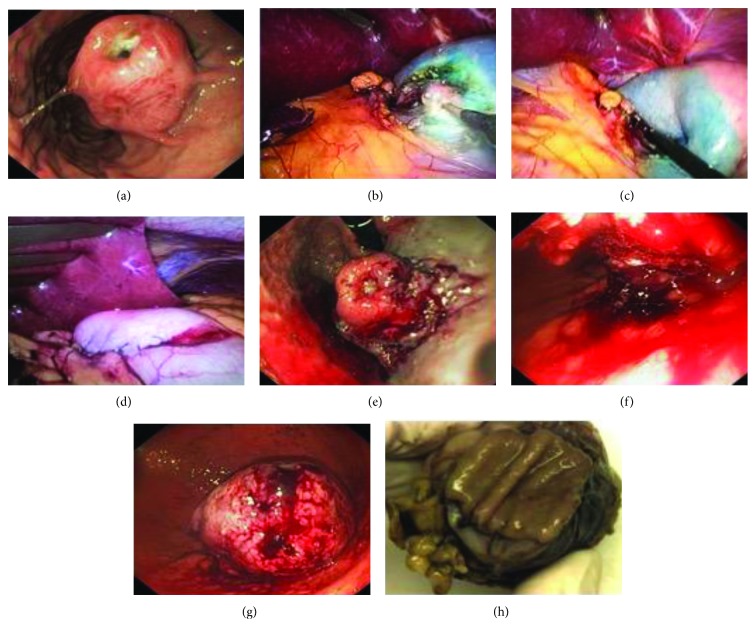
Nonexposed endoscopic wall-inversion surgery: intraoperative photos. (a) Endoscopic view of a gastrointestinal stromal tumour of the stomach. (b) Laparoscopic seromuscular incision around the lesion. (c) Inversion of the tumour into the stomach. (d) Laparoscopic suture. (e) Endoscopic muco-sub-mucosal incision. (f) Endoscopic muco-sub-mucosal dissection. (g) Resected specimen inside the stomach. (h) Resected specimen after endoscopic extraction.

**Figure 2:**
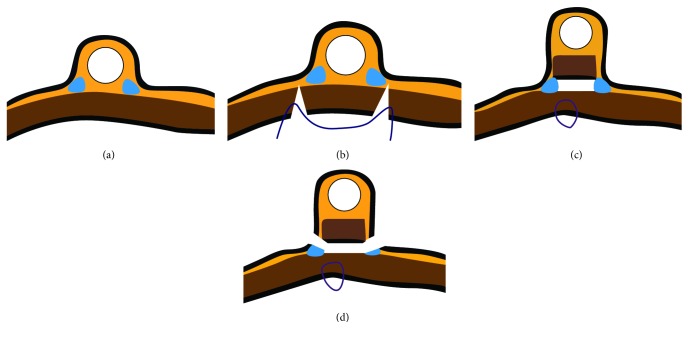
Nonexposed endoscopic wall-inversion surgery: illustration of the procedure. (a) Submucosal injection. (b) Seromuscular incision and laparoscopic suture. (c) Completion of the laparoscopic suture and inversion of the tumour in the gastric lumen. (d) Submucosal dissection.

All procedures were performed by a surgeon and an endoscopist. Intraoperative photographs are shown in Figure 1, and illustrations of the main steps of the procedure are shown in Figure 2. The patients were put under general anaesthesia and placed in the reverse Trendelenburg position. A 10 mm camera port was inserted above the umbilicus before a 12 mm Hg positive-pressure capnoperitoneum was produced. A laparoscopic four-quadrant examination was performed before introducing a 12 mm port in the right hypochondrium and a further two to three ports in the upper abdomen, depending on the location of the tumour. The endoscope was inserted, and the tumour was visualised on the internal surface of the stomach. The endoscopist used electrocautery (DualKnife, KD-650L; Olympus Medical Systems, Tokyo, Japan) to mark the extent of the resection including a safety margin. The next step was to inject a solution of saline, glycerol, and methylene blue into the resection margin via the endoscope. A laparoscopic hook was then used to dissect the seromuscular layer along the markings previously made on the serosa surface. The tumour was then inverted into the gastric lumen using traction from an endoscopic snare (Captivator 30 mm large oval snare, Boston Scientific) with simultaneous pressure from a laparoscopic grasper. After desufflating the stomach, a single layer of interrupted sutures (Vicryl 3/0) was used to close the defect. The DualKnife and ITknife2 (KD-611L; Olympus Medical Systems) were then used to dissect the submucosa, and the resection was completed. The tumour was extracted perorally with a Roth Net polyp retriever (US Endoscopy, Mentor, OH, USA). After the tumour was extracted, the integrity of the suture line was inspected both laparoscopically and endoscopically. An abdominal drain was inserted, laparoscopic ports were removed, and the capnoperitoneum was terminated. Patients were intubated with nasogastric tubes, which were left in situ for the first postoperative 24 hours. Sipping was commenced on the first postoperative day.

### 2.3. Endoscopic-Navigated Laparoscopic Wedge Resections

**Figure 3:**
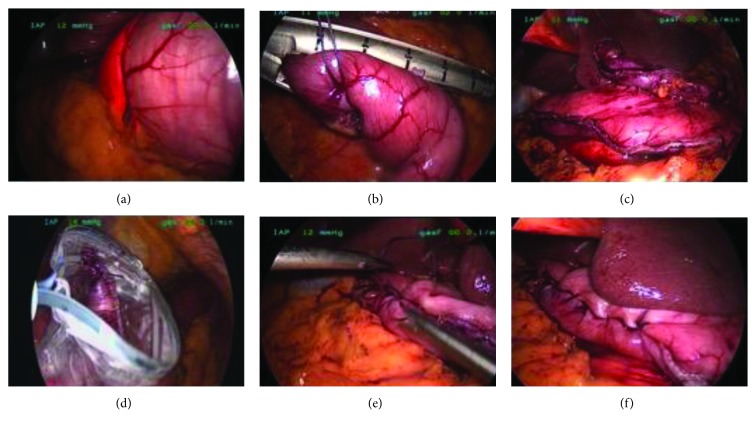
Endoscopic-navigated laparoscopic wedge resections: intraoperative photos. (a) Endoscopic and laparoscopic confirmation of the location of the tumour in the stomach. (b and c) Wedge resection performed with a stapler. (d) The resected specimen inserted into an endosack. (e and f) Suture of the staple line.

Intraoperative photographs of the main steps are shown in Figure 3. Under general anaesthesia, the patients were put in the reverse Trendelenburg position. An 11 mm port was placed above the umbilicus, and a 12 mm Hg positive-pressure capnoperitoneum was established. A four-quadrant inspection of the abdominal cavity was performed before introducing a 12 mm port in the right hypochondrium, a 12 mm port in the left hypochondrium, and an additional one or two ports in the epigastrium depending on the location of the tumour. The location of the tumour in the stomach was confirmed laparoscopically and endoscopically. Wedge resection was performed with a stapler (Endo GIA™ with Tri-Staple™ Technology; Medtronic, Minneapolis, USA) under endoscopic control. The staple line was reinforced with laparoscopic sutures. The resected specimen was inserted into an endosack and removed through the 12 mm port in the hypochondrium. The integrity of the suture line was inspected, an Easy-Flow or Robinson drain was inserted, and the capnoperitoneum was terminated. Patients were intubated with nasogastric tubes, which were left in situ for the first postoperative 24 hours. Sipping was commenced on the first postoperative day.

## 3. Results

The study group consisted of 11 patients who underwent NEWS, and the control group consisted of 12 patients who underwent LWR for gastric tumours. Results are summarised in Tables 1–3.

### 3.1. Patient Characteristics

#### 3.1.1. Study Group

The average patient age was 65 years with a range of 44 to 80. The average BMI was 27.8, and the male to female ratio was 7 : 4. Nine patients were asymptomatic; the tumours were discovered incidentally. One patient presented with anaemia and one with abdominal pain.

#### 3.1.2. Control Group

The average patient age was 65 years with a range of 31 to 77. The average BMI was 25.6, and the male to female ratio was 7 : 5. Five tumours were detected incidentally. Three presented with gastrointestinal bleeding, two with gastroesophageal reflux, one with dysphagia, and one with dyspeptic symptoms.

### 3.2. Tumour Characteristics

#### 3.2.1. Study Group

Three tumours were located in the subcardial region, two in the body, three in the fundus, and three in the prepyloric region. The average largest diameter of the tumours was 27 mm (range: 5 to 50 mm). The average difference in size between the largest diameter of the resected specimen and tumour was 13 mm. R0 resection margins were achieved in all cases.

Histological examination of the resected specimen revealed six GISTs, one submucosal lipoma, one leiomyoma, one endocrine tumour, one Vanek's tumour, and one case of ectopic pancreatic tissue. The GISTs were classified according to the NIH consensus statement. All had mitotic indices of less than 5 per 50 HPF and diameters of less than 50 mm and were thus classified as having very low malignant potential.

#### 3.2.2. Control Group

Five tumours were located in the body, four in the prepyloric region, two in the fundus, and one in the subcardial region. The average diameter of the tumours was 35 mm (range: 15 to 80 mm). The average difference in size between the largest diameter of the resected specimen and tumour was 30 mm. R0 resections margins were achieved in eleven of the 12 cases; in one case, tumour tissue was detected microscopically at the resection margin.

Histological examination revealed seven GISTs, one leiomyoma, two cases of ectopic pancreatic tissue, one endometriosis, and one hyperplasiogenic polyp. All seven GISTs had mitotic indices of less than 5 per 50 HPF. One had a diameter of 53 mm and was classified as having low malignant potential. The other six had diameters between 20 and 50 mm and were thus classified as having very low malignant potential.

### 3.3. Surgical and Perioperative Characteristics

#### 3.3.1. Study Group

The average operating time was 96 minutes (range: 70 to 120 ), and the average length of hospitalisation was 6.8 days (range: 5 to 10).

Two intraoperative complications occurred. In one case, bleeding occurred at the resection line after endoscopic submucosal dissection, which was successfully treated by endoclips (Figure 1(e)). In another case, bleeding occurred after the seromuscular incision, which was treated successfully by electrocoagulation.

Two postoperative complications occurred: one case of suture line bleeding and one subcapsular liver hematoma. The suture line bleeding presented as hematemesis. The patient underwent acute gastroscopy, which revealed resection line bleeding and was treated with argon photocoagulation and hemoclips. No significant drop in hemoglobin concentration occurred, and the patient remained stable throughout the postoperative period.

The subcapsular liver hematoma occurred in a patient with hepatic steatosis. The hematoma was presumably caused intraoperatively by the laparosopic retractor. It had a size of 64 × 67 mm and was drained under CT guidance in order to prevent abscess formation and to relieve the patient's pain. Postoperatively, the patient experienced pain in the right hypochondrium and so a computed tomography (CT) scan was performed.

#### 3.3.2. Control Group

The average length of operation was 62 minutes (range: 41 to 92). One case of suture line bleeding occurred, which was treated by the application of the hemostatic agent PerClot. No postoperative complications occurred. The average length of hospitalisation was 6.5 days (range: 3 to 11).

### 3.4. Follow-Up and Survival

#### 3.4.1. Study Group

The patients underwent follow-up gastroscopy at three and six months after surgery. At twelve months, they underwent gastroscopy with biopsy of the scar and endosonography. No tumour recurrences or gastric motility disorders were reported, and all patients remained alive and healthy throughout the follow-up period.

#### 3.4.2. Control Group

The patients underwent follow-up gastroscopy at three, six, and twelve months after surgery. Two deaths occurred (430 and 874 days after surgery), unrelated to the oncological disease. The one patient in whom R1 resection margins were reported remained recurrence-free throughout the follow-up period.

## 4. Discussion

Cooperative endoscopic-laparoscopic surgery for the resection of gastric tumours has been performed at the Department of Surgery of the Faculty Hospital Kralovske Vinohrady since January 2016. Our initial experience with these techniques was detailed in a previously published study [5]. The goal of this current study was to compare NEWS with LWR for the treatment of submucosal gastric tumours. We cooperated with the Department of Visceral Surgery of the Kepler University Hospital, a high-volume laparoscopic centre, to provide data on LWRs of gastric tumours.

After two reports on porcine models, NEWS was introduced by Mitsui et al. in 2014 in human patients [6, 7]. The initial report, based on six patients, was complicated by perforation of the stomach wall, which occurred in the first three cases [8]. This report was followed by a larger study on 20 patients by the same group of authors [9], in which perforation was reduced to 5%. The authors also reported negative resection margins in all cases, no recurrences and no problems with food intake in the follow-up period. In the current study, no perforation events occurred and similar clinical outcomes were achieved.

When NEWS was performed, dye was injected endoscopically into the submucosa, which could then be seen on the external surface of the stomach by the laparoscope. This allows for a more superior way of delineating the extent of the resection than relying solely on the endoscopic light shining through the gastric wall as is done in LWRs. We showed that the difference in size between the resected specimen and tumour was smaller and that negative resections were more frequently achieved in the NEWS patients. These more precise resections reduce the likelihood of stomach deformation from needless loss of excessive tissue, while still achieving negative resection margins. Furthermore, NEWS does not require opening the gastric wall, and therefore, gastric content (bacteria or tumour cells) does not contaminate the peritoneal cavity [3, 10].

Our NEWS technique differs slightly from the method described by Goto et al. [9] in that we omitted using a surgical spacer between the serosal side of the inverted lesion and the suture plane to facilitate the endoscopic submucosal dissection and prevent iatrogenic perforation. We found this step unnecessary; traction from the endoscopic snare with simultaneous pressure from the laparoscopic grasper was sufficient to invert the lesion. Additionally, by using the ITknife2 Electrosurgical Knife (KD-611L) (Olympus Medical Systems), which has an insulated tip and cuts the tissue laterally, we were able to avoid cutting through the sutures.

More precise resections achieved by NEWS may lead to a lower incidence of gastric motility disorders. At one year of follow-up no symptoms suggestive of gastric motility were reported in any of the patients who underwent NEWS. Similar results were achieved by Tsujimoto et al., who reported no evidence of gastric motility disorders in a cohort of 20 patients, and in a study by Waseda et al., who reported two cases of gastric motility disorders in a cohort of 22 patients [11, 12].

Despite these advantages to the hybrid approach, as these techniques are still in their early days there are some associated disadvantages thought to be due to limited experience and the technical difficulty of these techniques. The hybrid resections were associated with longer operation times when compared with standard laparoscopic resections. Although no serious complications occurred in either group, more complications were associated with the NEWS operations. Again, this reflects the complexity and limited experience of these operations and we expect the complication rate to decline with increased experience.

Length of hospitalisation was slightly longer in patients undergoing NEWS. Our main goal was to safely implement a new technique. Reduction of hospital stay is a secondary goal. The average length of hospital stay was increased by the two patients who had postoperative complications. When these patients are not included, the average length of stay was reduced to 6.3 days. We believe that with increasing experience the length of stay will decrease, and now we aim to discharge patients by the fifth postoperative day.

Tumour location, size, and direction of growth are the key factors when choosing the operative approach. Concerning size, we should emphasise that tumours with diameters larger than 4 cm can be resected using the NEWS technique as long as the other two diameters are under 4 cm, so tumour can be extracted via the oesophagus. Endoluminally growing tumours are more easily accessible by the endoscope and are more appropriate for the NEWS technique. Tumours in cardiac and pyloric regions are difficult for the laparoscopic approach and often necessitate open resections. These tumours can be more easily approached endoscopically and thus can be resected using the NEWS technique. In this study, three tumours in the cardiac region and two tumours in the pyloric region were successfully resected with the NEWS technique. When considering the size of the tumour, smaller tumours are more preferably resected using the NEWS technique. For larger tumours, laparoscopic or open wedge resection is more convenient.

## 5. Conclusion

Nonexposed endoscopic wall-inversion surgeries allow for more precise resections and more frequent achievement of negative resection margins than LWRs. They may result in better preservation of the gastric function and reduction of peritoneal contamination and tumour seeding. This study should be followed by larger prospective and randomised trials to verify our observations.

## Figures and Tables

**Table 1:** Patient characteristics.

	NEWS	LWR
Age (average, range)	65, 44-80	65, 31-77
Sex ratio (male : female)	7 : 4	7 : 5
BMI (average, range)	27.8, 17.9-38.7	25.6, 19.0-30.4
Presentation (symptomatic : asymptomatic)	2 : 9	7 : 5

**Table 2:** Tumour characteristics.

	NEWS	LWR
Location		
Subcardial region	**3**	**1**
*Anterior wall*	2	0
*Greater curvature*	1	0
*Angle of His*	0	1
Body	**2**	**5**
*Anterior wall*	0	5
*Posterior wall*	2	0
Fundus	**3**	**2**
*Anterior wall*	0	2
*Posterior wall*	2	0
*Fornix*	1	0
Prepyloric region	**3**	**4**
*Anterior wall*	1	4
*Posterior wall*	1	0
*Lesser curvature*	1	0

Diameter of resected specimen in mm (average, range)	40, 20-55	45, 35-95
Diameter of tumour in mm (average, range)	27, 5-50	35, 15-80
Difference in diameter between resected specimen and tumour in mm (average, range)	13, 5-23	30, 15-40

GIST tumours	6	7
Very low malignant potential	6	6
Low malignant potential	0	1
Other tumours	5	5

**Table 3:** Surgical and perioperative details.

	NEWS	LWR
Length of operation (minutes)	96, 70-120	62, 41-92
Intraoperative complications	2	1
Postoperative complications	2	0
Length of postoperative hospital stay (days)	6.8, 5-10	6.5, 3-11

## Data Availability

The data used to support the findings of this study are included within the article.

## References

[1] Ye L., Wu X., Wu T. (2017). Meta-analysis of laparoscopic vs. open resection of gastric gastrointestinal stromal tumors. *PLoS One*.

[2] Ohtani H., Maeda K., Noda E. (2013). Meta-analysis of laparoscopic and open surgery for gastric gastrointestinal stromal tumor. *Anticancer Research*.

[3] Maehata T., Goto O., Takeuchi H., Kitagawa Y., Yahagi N. (2015). Cutting edge of endoscopic full-thickness resection for gastric tumor. *World Journal of Gastrointestinal Endoscopy*.

[4] Fletcher C. D., Berman J. J., Corless C. (2002). Diagnosis of gastrointestinal stromal tumors: a consensus approach. *International Journal of Surgical Pathology*.

[5] Hajer J., Havluj L., Whitley A., Gurlich R. (2018). Non-exposure endoscopic-laparoscopic cooperative surgery for stomach tumors: first experience from the Czech Republic. *Clinical Endoscopy*.

[6] Goto O., Mitsui T., Fujishiro M. (2011). New method of endoscopic full-thickness resection: a pilot study of non-exposed endoscopic wall-inversion surgery in an ex vivo porcine model. *Gastric Cancer*.

[7] Mitsui T., Goto O., Shimizu N. (2013). Novel technique for full-thickness resection of gastric malignancy: feasibility of nonexposed endoscopic wall-inversion surgery (NEWS) in Porcine models. *Surgical Laparoscopy, Endoscopy & Percutaneous Techniques*.

[8] Mitsui T., Niimi K., Yamashita H. (2014). Non-exposed endoscopic wall-inversion surgery as a novel partial gastrectomy technique. *Gastric Cancer*.

[9] Goto O., Takeuchi H., Sasaki M. (2016). Laparoscopy-assisted endoscopic full-thickness resection of gastric subepithelial tumors using a nonexposure technique. *Endoscopy*.

[10] Ntourakis D., Mavrogenis G. (2015). Cooperative laparoscopic endoscopic and hybrid laparoscopic surgery for upper gastrointestinal tumors: current status. *World Journal of Gastroenterology*.

[11] Waseda Y., Doyama H., Inaki N. (2014). Does laparoscopic and endoscopic cooperative surgery for gastric submucosal tumors preserve residual gastric motility? Results of a retrospective single-center study. *PLoS One*.

[12] Tsujimoto H., Yaguchi Y., Kumano I., Takahata R., Ono S., Hase K. (2012). Successful gastric submucosal tumor resection using laparoscopic and endoscopic cooperative surgery. *World Journal of Surgery*.

